# Nanoparticle Aggregation and Thermophoretic Particle Deposition Process in the Flow of Micropolar Nanofluid over a Stretching Sheet

**DOI:** 10.3390/nano12060977

**Published:** 2022-03-16

**Authors:** Yangyang Yu, Javali K. Madhukesh, Umair Khan, Aurang Zaib, Abdel-Haleem Abdel-Aty, Ibrahim S. Yahia, Mohammed S. Alqahtani, Fuzhang Wang, Ahmed M. Galal

**Affiliations:** 1 School of Mathematics and Statistics, Xuzhou University of Technology, Xuzhou 221018, China; yyymath@126.com (Y.Y.); wangfuzhang1984@163.com (F.W.); 2 Department of Mathematics, Nanchang Institute of Technology, Nanchang 330044, China; 3 Department of Mathematics, Davangere University, Davangere 577002, India; madhukeshjk@gmail.com; 4 Department of Mathematical Sciences, Faculty of Science and Technology, University Kebangsaan Malaysia, UKM, Bangi 43600, Malaysia; umairkhan@iba-suk.edu.pk; 5 Department of Mathematics and Social Sciences, Sukkur IBA University, Sukkur 65200, Pakistan; 6 Department of Mathematical Sciences, Federal Urdu University of Arts, Science & Technology, Gulshan-e-Iqbal Karachi 75300, Pakistan; 7 Department of Physics, College of Sciences, University of Bisha, P.O. Box 344, Bisha 61922, Saudi Arabia; amabdelaty@ub.edu.sa; 8 Physics Department, Faculty of Science, Al-Azhar University, Assiut 71524, Egypt; 9 Laboratory of Nano-Smart Materials for Science and Technology (LNSMST), Department of Physics, Faculty of Science, King Khalid University, P.O. Box 9004, Abha 61413, Saudi Arabia; isyahia@gmail.com; 10 Research Center for Advanced Materials Science (RCAMS), King Khalid University, P.O. Box 9004, Abha 61413, Saudi Arabia; 11 Nanoscience Laboratory for Environmental and Biomedical Applications (NLEBA), Metallurgical Laboratory 1, Department of Physics, Faculty of Education, Ain Shams University, Roxy, Cairo 11757, Egypt; 12 Radiological Sciences Department, College of Applied Medical Sciences, King Khalid University, P.O. Box 9004, Abha 61421, Saudi Arabia; mosalqhtani@kku.edu.sa; 13 BioImaging Unit, Space Research Centre, Michael Atiyah Building, University of Leicester, Leicester LE1 7 RH, UK; 14 Mechanical Engineering Department, College of Engineering, Prince Sattam Bin Abdulaziz University, Wadiaddawaser 11991, Saudi Arabia; ahm.mohamed@psau.edu.sa; 15 Production Engineering and Mechanical Design Department, Faculty of Engineering, Mansoura University, Mansoura 35516, Egypt

**Keywords:** micropolar nanofluid, nanoparticle aggregation, heat source/sink, thermophoretic particle deposition, bioconvection

## Abstract

The purpose of this research is to investigate the consequence of thermophoretic particle deposition (TPD) on the movement of a TiO_2_/water-based micropolar nanoliquid surface in the existence of a porous medium, a heat source/sink, and bioconvection. Movement, temperature, and mass transfer measurements are also performed in the attendance and nonappearance of nanoparticle aggregation. The nonlinear partial differential equations are transformed into a system of ordinary differential equations using appropriate similarity factors, and numerical research is carried out using the Runge-Kutta-Felhberg 4th/5th order and shooting technique. The obtained results show that improved values of the porous constraint will decline the velocity profile. Improvement in heat source/sink parameter directly affects the temperature profile. Thermophoretic parameter, bioconvection Peclet number, and Lewis number decrease the concentration and bioconvection profiles. Increases in the heat source/sink constraint and solid volume fraction will advance the rate of thermal dispersion. Nanoparticle with aggregation exhibits less impact in case of velocity profile, but shows a greater impact on temperature, concentration, and bioconvection profiles.

## 1. Introduction

Non-Newtonian materials are frequently encountered in a plethora of technological applications, including the production of crude soft elastic materials, the behavior of lubricants, sludges, pigments, packaged foods, and the mobility of organic fluids. Rheological fluids include blood, sauces, solvent cosmetics, colors, some lubricants, and rupturing mud. The relationship between stress and strain within those fluids is nonlinear. These liquids are substantially more complex to study than Newtonian fluids. As a result, various nonlinear frameworks for non-Newtonian liquids have been proposed. Even Navier–Stokes formulas are complex, containing a lot of restrictions, and the findings of resulting equalities are more difficult to acquire [1]. Eringen [2] shows that the Navier–Stokes theory fails to precisely describe the properties of different liquids indicated by microscopic characteristics arising from the micromotions and local structure of liquid constituents. These fluids are used to investigate the properties of contaminated lubricants, oils, polymeric mixtures, nanocrystals, animal blood containing rigid cells, and a variety of other biological solutions. Based on the wide range of applications, many researchers are working on this fluid model recently are addressed here [3,4,5,6,7,8].

Nanofluids have a wide range of roles in industry and engineering. The use of nanoparticles in fluid flow analysis has shown several uses such as power generation, microscale electronics, chemical processes, etc. Depending on the type of nanoparticles used in the composition procedure, nanofluid is also known as nanomaterials. Improved heat conductivity, which is the core and fundamental property of nanomaterials, is crucial. The most often used base fluids in this regard are water, toluene, ethylene, and kerosene oil, and several related research have recently been published to actually apply the concept of nanofluids. Nanofluids have distinct properties that make them particularly useful in a variety of thermal control activities. They have better thermal conductivity as well as a better convective heat transfer coefficient than the base fluid. Bahiraei et al. [9,10] examined the heat transport and entropy analysis in the presence of nanofluid. Khan et al. [11] explored the unstable stagnation point motion of hybrid nanoparticles over a spinning disk. The impacts of a magnetic dipole in the attendance of the KKL model to stimulate radiative nano liquid flow across a stretched sheet via Kumar et al. [12] Alhadhrami et al. [13] studied the effect of TPD on nanoliquid Glauert wall jet slip flow. In the existence of chemically reactive activation energy, Madhukesh et al. [14] examined the Marangoni-Bio convection movement of Casson nano liquid across a porous media. Muhammad and Nadeem [15] discussed convective heat transfer in the presence of magnetic dipole by using nickelzinc ferrite, manganese zinc ferrite, and magnetite ferrite with ethylene glycol. Guo et al. [16] examined the heat transfer characteristics in a nanofluid containing γ-Fe_2_O_3_ nanoparticles. Xie et al. [17] studied thermal conductivity improvement in aluminum nanoparticles suspended in a base fluid. They found that thermal conductivity is improved with the suspension of nanoparticles. The researchers have always found it difficult to model the thermophysical parameters in such a way that they are consistent with the experimental findings. Using standard models, the gain in the thermal conductivity of nanoliquids was grossly overestimated. The relative viscosity of aggregated nanoparticles was modeled using the fractal approach [18]. Recently, Mackolil and Mahanthesh [19] investigated the effects of nanoparticle aggregation and temperature-dependent surface tension on Marangoni convection in TiO_2_–EG nanoliquid. Here are some of the most important papers on nanoparticle aggregation [20,21,22].

Thermal transportation and dynamics of fluid flow research have scientific and practical applications in cable design, plastic sheet engineering, fiberglass structure, and thermal spouting. The heat source/sink (HS/S) is another major factor that determines heat transmission. When a heat source or heat sink is used, the heat dispersal throughout the entire field changes. In this view, many researchers do significant works on HS/S. Recently, Saleh et al. [23] probed the magnetic dipole and HS/S effects on Maxwell hybrid nanofluid flow across a stretched sheet. Madhukesh et al. [24] inspected the behavior of water transporting SWCNT nanoparticles and swimming microorganisms across a Riga plate with an HS/S. Micropolar liquid moving through a convectively hot surface, containing an nth order chemical process and an HS/S, was studied by Sajid et al. [25]. Khan et al. [26] investigated the mixed convection movement of pair stress nanofluid with HS/S effects across an oscillating stretchable surface. Chu et al. [27] looked at mathematical simulations of time-dependent micro-rotation blood circulation induced by a curved moving surface via gold nanoparticle conduction with non-uniform HS/S.

When there is a temperature gradient, the suspended particle will tend to travel from high to low-temperature areas. The force that causes this phenomenon is known as the thermophoretic force. The phenomenon of thermophoresis is important in the mass transfer mechanism of numerous devices that use microscopic micron-sized particles and huge temperature differences in the fields. Many researchers have shown interest in this concept over various geometries. Shehzad et al. [28] examined the forced convective Maxwell fluid flow across a spinning disc while thermophoretic particles motion. Kumar et al. [29] investigated the effects of thermophoretic particle’s deposition (TPD) on heat and mass transfer in Casson fluid flow through a moving thin needle. Some of the works on the notion of TPD can be found in [30,31,32].

Microbes are monocellular organisms that are significantly more productive than plants at lessening the greenhouse gases and absorption of CO_2_ from the environment. Monocellular organisms, on the other hand, can only be employed in a mixture of nanofluids. The fertility of the soil is improved by using microorganisms. Microbes, such as shielded cells, are considered as gyrotactic microorganisms for moving to a liquid for increasing density stratification based on the gradient thickness. Gyrotactic microorganisms are added to nanofluids to promote nanoparticle preservation and mass propagation. Ali et al. [33] investigated the influence of combined convection and magnetohydrodynamic (MHD) flow on the dynamics of Casson nano liquid in the stagnation point of a revolving sphere with finite element modelling. Alqarni et al. [34] evaluated the impact of bioconvection in 3D viscoelastic nanoliquid movement due to exponentially stretched surface with nonlinear radiative heat transmission and variable thermal conductivity. Revised Fourier’s and Fick’s Laws for duplicating mixed bioconvective motion of radiative-reactive Walters-B liquid transmission of minute particles subject to Lorentz force on radiative-reactive Walters-B fluids were investigated by Wakif et al. [35]. Farooq et al. [36] investigated the thermally radioactive bioconvection current of Carreau nano liquid using modified Cattaneo–Christov expressions and an exponential space-based heat source.

According to the previous section affordable literature study, no study on micropolar nanoparticles flow across a stretched sheet in the existence of the porous media, HS/S, TPD, and bioconvection has been done. The present paper is presented to study the influence of nanoparticle aggregation and without aggregation on various parameters over respective profiles. The governing equations are converted into a system of ODEs and solved numerically. The influence of various parameters is studied in detail. The current examination is conducted to obtain the answers to the following questions.

- What is the influence of nanoparticles with and without aggregation on porosity parameter over a velocity profile?
- What is the impact of nanoparticles with and without aggregation over a thermal distribution?
- What is the effect of HS/S parameter, thermophoretic parameter, bioconvection Peclet number and bioconvection Lewis number on respective profiles?

## 2. Mathematical Formulation

**Figure 1:**
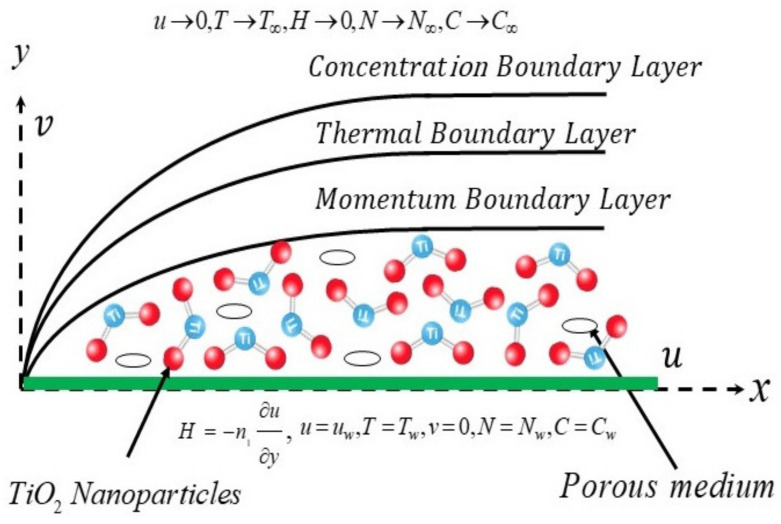
Physical model of the problem.

The steady, laminar, incompressible boundary layer flow of a micropolar nanofluid movement over a continuous stretching sheet in the presence of the porous medium, HS/S, TPD and bioconvection is considered. The flow configuration of the model is schematically revealed in Figure 1, where x and y represents the respective Cartesian coordinate axes. The x− axis is taken along the stretching surface and y− axis is measured normal to it. Therefore, the wall uniform stretching velocity of the sheet is specified by $u_{w}=ax$, where a is a positive constant. In addition, the constant temperature of the fluid at the wall and ambient scenario are represented via $T_{w}$ and $T_{\infty }$, respectively. The constant concentration and motile microorganism at the wall surface of the sheet is dignified via $C_{w}$ and $N_{w}$, respectively, while $C_{\infty }$ and $N_{\infty }$ denoted the respective uniform ambient concentration and the motile microorganism. Under these requisite posited assumptions, the governing equations for continuity, momentum, temperature, concentration and bioconvection in terms of PDEs are stated as (see, e.g., in [37,38,39]).

(1) $$\frac{\partial u}{\partial x}+\frac{\partial v}{\partial y}=0,$$

(2) $$u\frac{\partial u}{\partial x}+v\frac{\partial u}{\partial y}=-\frac{K_{1}}{\rho_{nf}}\frac{\partial H}{\partial y}-\frac{\upsilon_{nf}}{K^{*}}u+\frac{\partial^{2}u}{\partial y^{2}}\left(\left(K_{1}+\mu_{nf}\right)\frac{1}{\rho_{nf}}\right),$$

(3) $$u\frac{\partial H}{\partial x}+v\frac{\partial H}{\partial y}=\frac{\partial^{2}H}{\partial y^{2}}\left(\frac{\Omega_{1}}{\rho_{nf}j}\right)-\left(2H+\frac{\partial u}{\partial y}\right)\frac{K_{1}}{\rho_{nf}j},$$

(4) $$u\frac{\partial T}{\partial x}+v\frac{\partial T}{\partial y}=\frac{k_{nf}}{{\left(\rho C_{p}\right)}_{nf}}\frac{\partial^{2}T}{\partial y^{2}}+\frac{Q_{1}}{{\left(\rho C_{p}\right)}_{nf}}\left(T-T_{\infty }\right),$$

(5) $$u\frac{\partial C}{\partial x}+v\frac{\partial C}{\partial y}=D_{B}\frac{\partial^{2}C}{\partial y^{2}}-\frac{\partial }{\partial y}\left(V_{T}\left(C-C_{\infty }\right)\right),$$

(6) $$u\frac{\partial N}{\partial x}+v\frac{\partial N}{\partial y}=D_{m}\frac{\partial^{2}N}{\partial y^{2}}-\frac{\partial }{\partial y}\left(\frac{bWc}{C_{w}-C_{\infty }}\left(N\frac{\partial C}{\partial y}\right)\right),$$

along with subject to the boundary conditions

(7) $$u=u_{w},\;\;v=0,\;\;N=N_{w},\;H=-n_{1}\frac{\partial u}{\partial y},\;\;T=T_{w},\;C=C_{w}\;\mathrm{at}\;y=0,$$

(8) $$u\to 0,\;\;T\to T_{\infty },\;\;N\to N_{\infty },H\to 0,\;C\to C_{\infty }\;\mathrm{as}\;y\to \infty .$$

Furthermore, the spin gradient viscosity is given by $\Omega_{1}=\mu_{f}\left(\frac{\mu_{nf}}{\mu_{f}}+\frac{\alpha_{1}}{2}\right)j\to \left(\mu_{nf}+\frac{K_{1}}{2}\right)j$ (see, e.g., in [31]), the micropolar parameter is given as $\alpha_{1}=\frac{K_{1}}{\mu_{f}}$ and $j=\frac{\upsilon_{f}}{a}$ signifies the reference length. The microrotation parameter is denoted by $n_{1}$, where $n_{1}\in \left[0,1\right]$. In the absence of microrotation parameter, the microelements are highly concentrated and unable to spin close to the wall surface.

According to the work in [32], the thermophoretic velocity is demarcated as

(9) $$V_{T}=-\frac{\upsilon_{f}K_{2}}{T_{r}}\frac{\partial T}{\partial y}$$

where $K_{2}$ and $T_{r}$ are the thermophoretic constant and reference temperature, respectively.

According to experimental and empirical investigations (see, e.g., in [19,40,41]), the nanoparticle aggregation factor is important in the dynamics and heat transmission of nanofluid flows. When the aggregation component was taken into consideration, the measurement results of the nanomaterial agreed precisely. With the aggregation kinetic factor, the nanoparticle volume fraction becomes

(10) $$\phi_{a1}=\phi {\left(\frac{r_{a1}}{r_{p1}}\right)}^{3-D_{1}}.$$

The thermal conductivity is computed by combining the Bruggeman and modified Maxwell models, as indicated in Table 1 (see [42]) and the appropriate expressions are provided below (see, e.g., in [43,44,45]):

(11) $$\frac{k_{a1}}{k_{f}}=0.25\left\{\begin{array}{l} \frac{k_{p1}}{k_{f}}\left(3\phi_{i1}-1\right)+\left(3\left(1-\phi_{i1}\right)-1\right)+\\ {\left[{\left(\frac{k_{p1}}{k_{f}}\left(3\phi_{i}{}_{1}-1\right)+\left(3\left(1-\phi_{i1}\right)-1\right)\right)}^{2}+8\frac{k_{p1}}{k_{f}}\right]}^{0.5} \end{array}\right\},$$

(12) $$\phi_{i1}={\left(\frac{r_{a1}}{r_{p1}}\right)}^{D_{1}-3},$$

(13) $$\rho_{a1}=\left(1-\phi_{i1}\right)\rho_{f}+\phi_{i1}\rho_{s},$$

(14) $${\left(\rho C_{p}\right)}_{a1}=\left(1-\phi_{i1}\right){\left(\rho C_{p}\right)}_{f}+\phi_{i1}{\left(\rho C_{p}\right)}_{s}.$$

From Equations (12)–(14), the subscript a1 denotes aggregates and p1 signifies nanoparticles. Fractional index $D_{1}=1.8$, $\frac{r_{a1}}{r_{p1}}=3.34$, $\phi_{m1}=0.605$ reflect fast-moving flows and about the mono disperse system are the commonly accepted values.

Furthermore, the similarity transformations for the considered requisite problem are defined as

(15) $$\left\{\begin{array}{l} \Psi =xf\left(\eta \right)\sqrt{a\upsilon_{f}},\;u=\frac{\partial \Psi }{\partial y},\;\;v=-\frac{\partial \Psi }{\partial x},\;H=ax\sqrt{\frac{a}{\upsilon_{f}}}g\left(\eta \right),\\ \eta =\sqrt{\frac{a}{\upsilon_{f}}}y,\;\theta \left(\eta \right)=\frac{T-T_{w}}{T_{w}-T_{\infty }},\;\varphi \left(\eta \right)=\frac{N-N_{w}}{N_{w}-N_{\infty }},\;\chi \left(\eta \right)=\frac{C-C_{w}}{C_{w}-C_{\infty }}. \end{array}\right.$$

Implementing the Equation (15) into leading governing equations, we get the following reduced form of similarity equations:

(16) $$f^{\prime \prime \prime }\left(\frac{1}{A_{1}}+\alpha_{1}\right)+A_{2}\left({ff}^{\prime \prime }-f^{\prime 2}\right)+\alpha_{1}g^{\prime }-\frac{\beta_{1}}{A_{1}}f^{\prime }=0,$$

(17) $$g^{\prime \prime }\left(\frac{1}{A_{1}}+\frac{\alpha_{1}}{2}\right)+A_{2}\left({fg}^{\prime }-f^{\prime }g\right)-\alpha_{1}\left(2g+f^{\prime \prime }\right)=0,$$

(18) $$\frac{k_{hnf}}{k_{f}}\theta^{\prime \prime }+A_{3}\mathrm{Pr}f\theta^{\prime }+\mathrm{Pr}Hs\theta =0,$$

(19) $$\frac{\chi^{\prime \prime }}{Sc}+f\prime^{-}\chi \tau \left(\theta^{\prime \prime }\chi +\theta^{\prime }\chi^{\prime }\right)=0,$$

(20) $$\varphi^{\prime \prime }+Lbf\varphi^{\prime }-Pe\left(\chi^{\prime }\varphi^{\prime }+\chi^{\prime \prime }(\sigma +\varphi )\right)=0,$$

with subject transformed BCs:

(21) $$f\left(0\right)=0,\;\;g\left(0\right)=-n_{1}f^{\prime \prime }\left(0\right),\;\;\theta \left(0\right)=\chi \left(0\right)=\varphi \left(0\right)=f^{\prime }\left(0\right)=1,\;\mathrm{at}\;\eta =0,$$

(22) $$f^{\prime }\left(\eta \right)\to 0,\;\;\theta \left(\eta \right)\to 0,\;\;\varphi \left(\eta \right)\to 0,g\left(\eta \right)\to 0,\;\chi \left(\eta \right)\to 0\;\mathrm{as}\;\eta \to \infty .$$

Similarly, Equations (16)–(22) comprised distinct controlling parameters which are namely and symbolically given as follows:$\beta =\upsilon_{f}/aK^{*}$ signifies porous constraint, $Hs=Q_{1}/a{\left(\rho C_{p}\right)}_{f}$ signifies heat source/sink constraint, $\mathrm{Pr}=\upsilon_{f}/\alpha_{f}$ signifies Prandtl number, $Sc=\upsilon_{f}/D_{B}$ signifies Schmidt number, $\tau =-K_{2}\left(T_{w}-T_{\infty }\right)/T_{r}$ signifies thermophoretic constraint, $Lb=\upsilon_{f}/D_{m}$ signifies bioconvection Lewis number, $Pe=bWc/D_{m}$ signifies bioconvection Peclet number, $\sigma =N_{\infty }/N_{w}-N_{\infty }$ signifies concentration difference parameter and the other thermophysical constants expressions are demarcated as

$A_{1}={\left(1-\frac{\phi_{a1}}{\phi_{m1}}\right)}^{2.5\;\;\phi_{m1}}$, $A_{2}=\left(1-\phi_{a1}+\phi_{a1}\frac{\rho_{a1}}{\rho_{f}}\right)$ and $A_{3}=\left(1+\frac{{\left(\rho Cp\right)}_{a1}}{{\left(\rho Cp\right)}_{f}}\phi_{a1}-\phi_{a1}\right)$.

The important engineering factors like Skin friction, wall couple stress factor, Nusselt number, Sherwood number and density number of motile microorganisms is given as

(23) $$\left.\begin{array}{l} C_{f}=\frac{\tau_{w}}{\rho_{f}u_{w}^{2}},C_{s}=\frac{M_{w}}{\mu_{f}u_{w}},Nu_{x}=\frac{xq_{w}}{k_{f}\left(T_{w}-T_{\infty }\right)}\\ Sh_{x}=\frac{xq_{m}}{D_{B}\left(C_{w}-C_{\infty }\right)},Nn_{x}=\frac{xq_{n}}{D_{m}\left(N_{w}-N_{\infty }\right)} \end{array}\right\}$$

The terms $\tau_{w},M_{w},q_{w},q_{m},\;\mathrm{and}\;q_{n}$ defined as

(24) $$\tau_{w}=\left(\mu_{nf}+K_{1}\right){\left.\frac{\partial u}{\partial y}+K_{1}H\right|}_{y=0}$$

(25) $$M_{w}={\left.\frac{\partial H}{\partial y}\right|}_{y=0}\times \left(\mu_{nf}+\frac{K_{1}}{2}\right)j$$

(26) $$q_{w}=-{\left.\frac{\partial T}{\partial y}\right|}_{y=0}\times \left(k_{nf}\right)$$

(27) $$q_{m}=-{\left.\frac{\partial C}{\partial y}\right|}_{y=0}\times \left(D_{B}\right)$$

(28) $$q_{n}=-D_{m}{\left.\frac{\partial N}{\partial y}\right|}_{y=0}$$

Using Equations (15) and (21) the above expressions reduced as follows:

(29) Cf=1A1+α11−n1f″0Re,Cs=1A1+α12g′0,Nux=−knfkfθ′0Re,Shx=−Reχ′0,Nnx=−Reφ′0.

## 3. Numerical Process and Authentication of Code

The RKF-45 scheme and the procedure of shooting are exercised to tackle the set of Equations (16)–(20) and BCs (21) and (22). Because the resultant equations are of higher order and have two points. We must first convert this to an initial value concern in order to solve it. Let us consider the new variables:

$$\begin{array}{l} f=h1_{1},f^{\prime }=h1_{2},f^{\prime \prime }=h1_{3},f^{\prime \prime \prime }=h1_{3}^{\prime },\\ g=h1_{4},g^{\prime }=h1_{5},g^{\prime \prime }=h1_{5}^{\prime },\\ \theta =h1_{6},\theta^{\prime }=h1_{7},\theta^{\prime \prime }=h1_{7}^{\prime },\\ \chi =h1_{8},\chi^{\prime }=h1_{9},\chi^{\prime \prime }=h1_{9}^{\prime },\\ \varphi =h1_{10},\varphi^{\prime }=h1_{11},\varphi^{\prime \prime }=h1_{11}^{\prime }. \end{array}$$

(30) $$h_{3}^{\prime }=-\left(A_{2}\left(h1_{1}h1_{3}-{\left(h1_{2}\right)}^{2}\right)+\alpha_{1}h1_{5}-\frac{\beta }{A_{1}}h1_{2}\right)/\left(\frac{1}{A_{1}}+\alpha_{1}\right)$$

(31) $$h_{5}^{\prime }=-\left(A_{2}\left(h1_{1}h1_{5}-h1_{2}h1_{4}\right)-\alpha_{1}\left(2h1_{4}+h1_{3}\right)\right)/\left(\frac{1}{A_{1}}+\frac{\alpha_{1}}{2}\right)$$

(32) $$h_{7}^{\prime }=-\left(A_{3}h1_{1}h1_{7}+Hsh1_{6}\right)/\left(\frac{k_{nf}}{k_{f}\mathrm{Pr}}\right)$$

(33) $$h_{9}^{\prime }=-Sc\left(h1_{1}h1_{9}-\tau \left(h1_{7}^{\prime }h1_{8}+h1_{7}h1_{9}\right)\right)$$

(34) $$h_{11}^{\prime }=-\left(Lbh1_{1}h1_{11}-Pe\left(h1_{9}h1_{11}+h1_{9}^{\prime }(\sigma +h1_{10})\right)\right).$$

with

(35) $$\left.\begin{array}{l} h1_{1}\left(0\right)=0,h1_{2}\left(0\right)=1,h1_{3}\left(0\right)=-h1_{4}\left(0\right)/n_{1},\\ h1_{4}\left(0\right)=-n_{1}h1_{3}\left(0\right),h1_{5}\left(0\right)=\gamma_{1},\\ h1_{6}\left(0\right)=1,h1_{7}\left(0\right)=\gamma_{2},\\ h1_{8}\left(0\right)=1,h1_{9}\left(0\right)=\gamma_{3},\\ h1_{10}\left(0\right)=1,h1_{11}\left(0\right)=\gamma_{4}. \end{array}\right\}$$

The transformed initial value problem Equations (30)–(35)are numerically solved by using the thermo-physical characteristics of nanofluid provided in Table 1 (see [42]) and thermophysical properties of nanomaterial and base fluid given in Table 2 (see [38]) by varying the parameter values, $\alpha_{1}=0.1$, β=0.1, Hs=0.5, Sc=0.8, τ=0.1, Lb=Pe=1, σ=0.1, and $n_{1}=1$ quantitatively solved by estimating the unknowns using the shooting approach. With a step size of 0.1 and an acceptance inaccuracy of approximately $10^{-8}$. The numerical results of the present study have been compared with results of previous literature [46,47,48] and found to be the better match (see Table 3).

## 4. Analysis of Results

This section outlines that how the numerous regulating factors determine velocity and temperature profiles, concentration, motile microorganism profiles, and engineering coefficients are examined in greater details. The analysis is conducted in two modes namely, (1) with aggregation $\phi_{i1}\neq 1$, and (2) without aggregation over a porous, micro- polar constraint, heat sink/source constraint, thermophoretic constraint, bio-convection Lewis number and bio-convection Peclet number.

**Figure 2:**
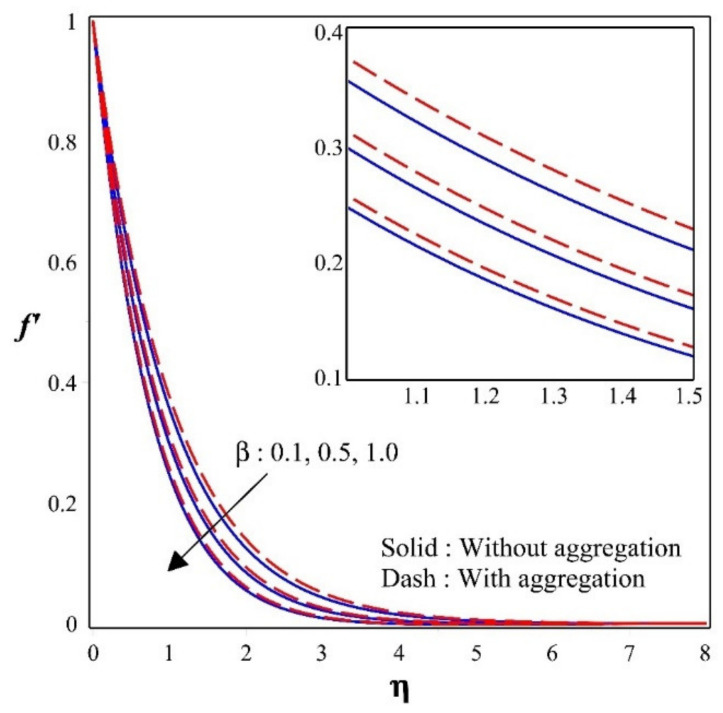
Sway of $f^{\prime }$ for different values of β.

Figure 2 illustrates the effect of porous parameter β on a dimensionless velocity profile $f^{\prime }$. Inclination in the values of β will declines $f^{\prime }$. This is because that the drag force faces the profiles of velocity movement in the existence of porous space, causing the speed to decline. Moreover, the diagram illustrates that in the absence of nanoparticle aggregation, the fluid velocity is lower than in the presence of nanoparticle aggregation.

**Figure 3:**
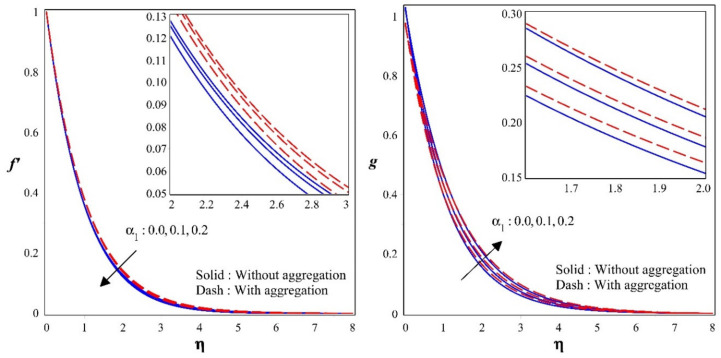
Sway of f′&g for different values of $\alpha_{1}$.

Figure 3 demonstrations the influence of micropolar constraint $\alpha_{1}$ over a velocity $f^{\prime }$ and micro rotation velocity fields g. Further, noted from the figure that when the micropolar constraint $\alpha_{1}$ is small, the viscous impact is limited to a very thin layer close to the wall, and the thickness of the boundary layer is equivalent to $\alpha_{1}$ and inversely relative to micro rotation velocity g. Upgrading in the values of $\alpha_{1}$ will declines the velocity profile but escalates the micro rotation velocity form. It is also observed that velocity is more in the presence of aggregation than absence of aggregation in $f^{\prime }$ but, reverse trend is seen in g.

**Figure 4:**
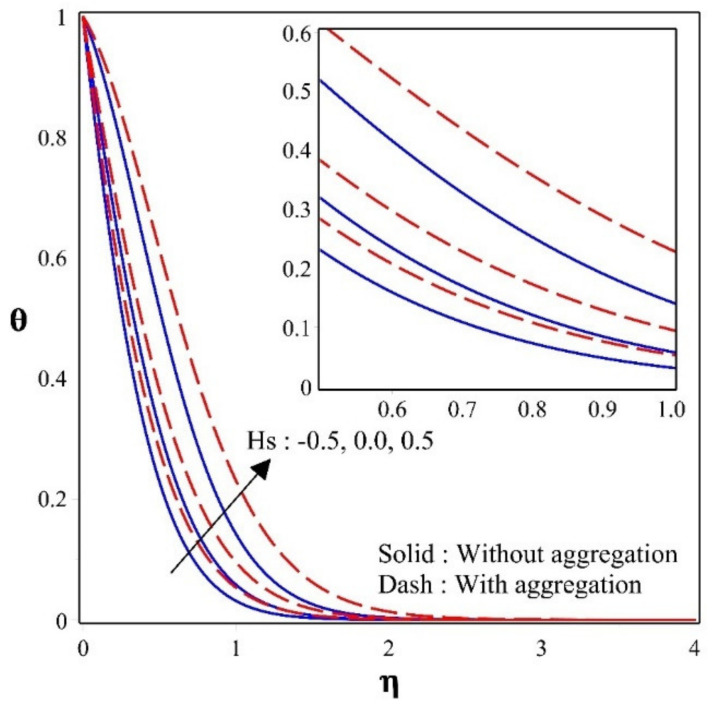
Sway of θ for different values of Hs.

The discrepancy of HS/S constraint over a temperature profile is illustrated in the Figure 4. Where heat dispersion is a major critical factor in engineering, this profile plays a vital role. Up gradation in the HS/S parameter will enhance the thermal dispersal in the given system. The heat sink will behave like a functional as a confine changer, transferring the heat created by the body into the nanoliquid. Consequently, in a given circumstance of a HS, the thermal dispersion is diffident, but in the occasion of a heat source, the heat is created by the body surface. In the occurrence of a heat source, higher thermal act is seen than in the absence of a heat sink. The graphic clearly shows that heat dispersion in nanoparticles with aggregation is greater than in nanoparticles without aggregation.

**Figure 5:**
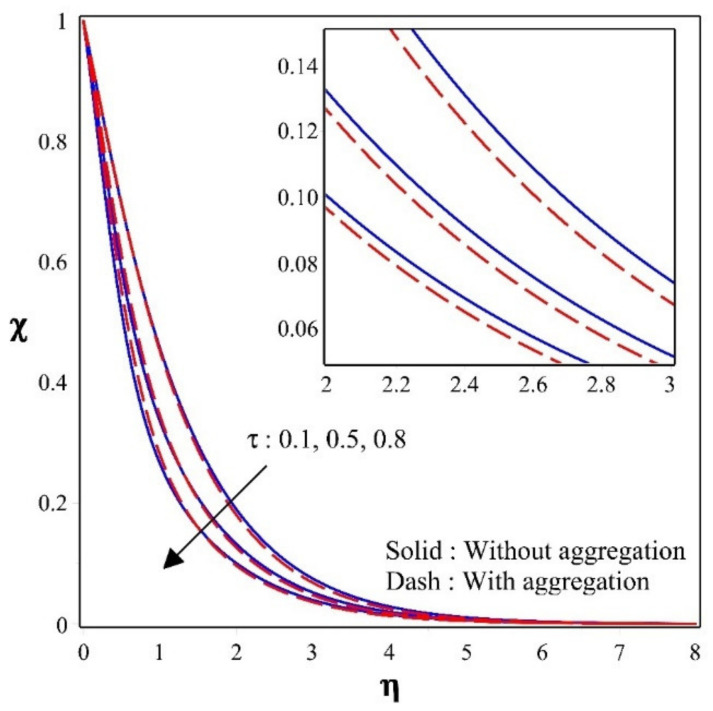
Sway of χ for different values of τ.

Figure 5 shows the discrepancy of thermophoretic constraint over a concentration profile. Escalating values of will diminishes the concentration. The motion of the nanoparticles enhances as the temperature gradient rises, ensuing in a lower concentration. The figure also shows that nanoparticles with aggregation have a lower concentration than nanoparticles without aggregation.

**Figure 6:**
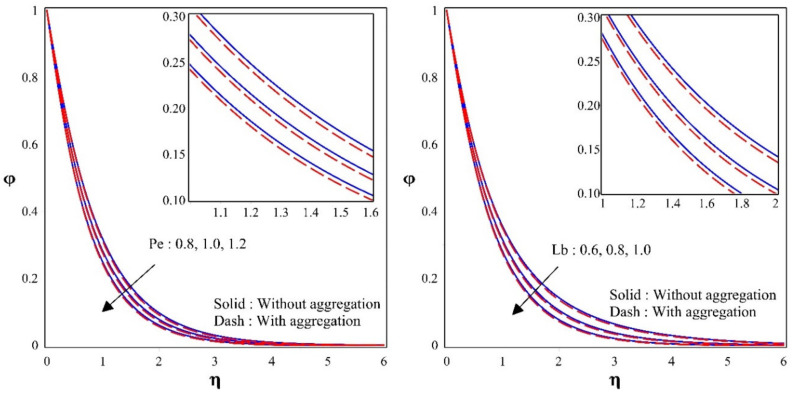
Sway of φ for different values of Pe&Lb.

Figure 6 illustrates the variation of bioconvection Peclet number Pe and bioconvection Lewis number Lb over bioconvection profile φ. Rise in the values in Pe and Lb will decreases the φ. The percentage of thermal diffusivity with mass diffusivity is known as the Lewis number. It is sometimes stated as a ratio between the Schmidt number and the Prandtl number. Pe and Lb results in decrease in microorganism dispersion, while density and BLT for motile microorganisms are decreasing. The figure also shows that nanoparticles with aggregation have a lower bioconvection profile than nanoparticles without aggregation.

**Figure 7:**
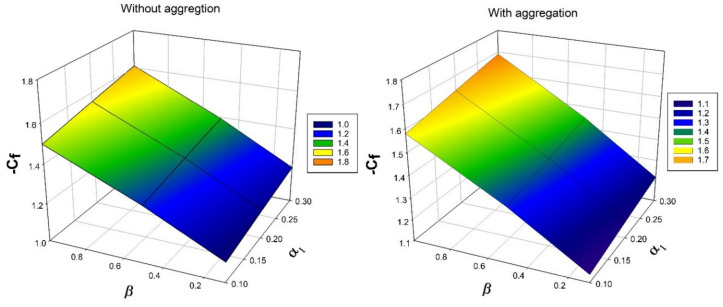
Nature of Skin friction $\left(-Cf\right)$ over Micropolar parameter $\left(\alpha_{1}\right)$ for numerous values of porous parameter $\left(\beta \right)$.

**Figure 8:**
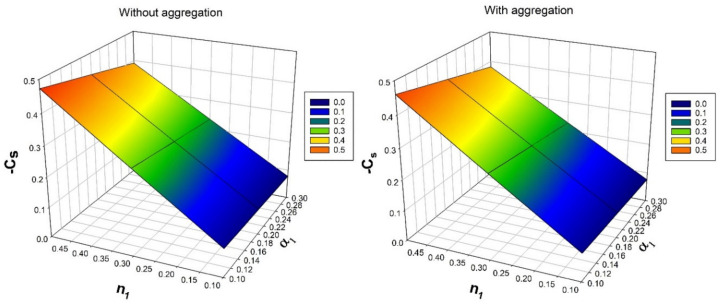
Nature of wall couple stress factor $\left(-Cs\right)$ over Micropolar parameter $\left(\alpha_{1}\right)$ for numerous values ofmicrorotation parameter $\left(n_{1}\right)$.

**Figure 9:**
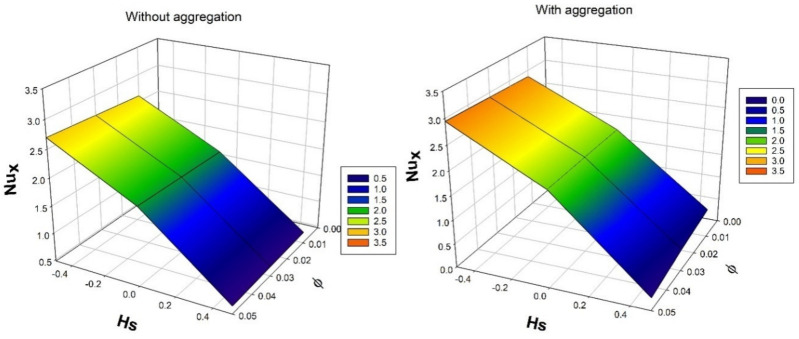
Nature of Nusselt number $\left(Nu_{x}\right)$ over solid volume fraction $\left(\phi \right)$ for numerous values of heat source/sink parameter $\left(Hs\right)$.

**Figure 10:**
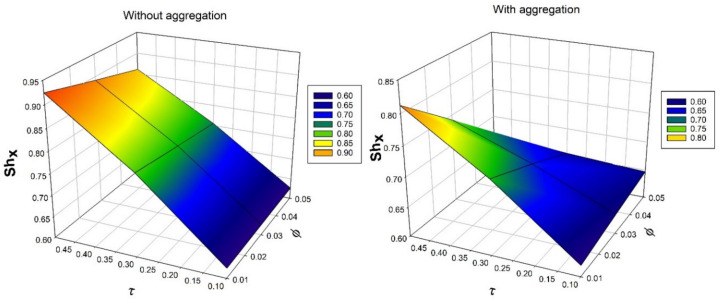
Nature of Sherwood number $\left(Sh_{x}\right)$ over solid volume fraction $\left(\phi \right)$ for numerous values of thermophoretic parameter $\left(\tau \right)$.

**Figure 11:**
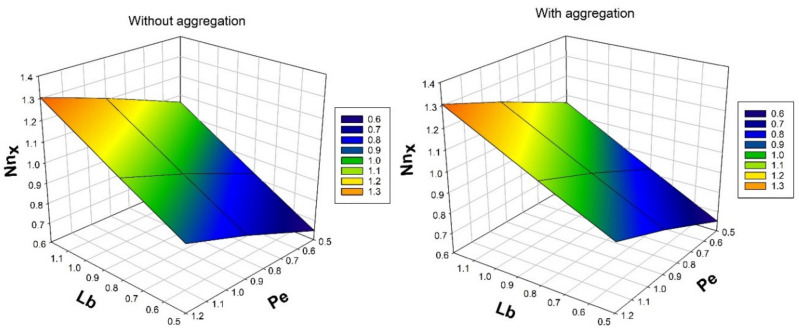
Nature of density number of motile microorganisms $\left(Nn_{x}\right)$ over bioconvection Peclet number Pe for numerous values of bioconvection Lewis number Lb.

Figure 7, Figure 8, Figure 9, Figure 10 and Figure 11 show the effect of several dimensionless constraints on important primary engineering coefficients. Figure 7 depicts the effect of micropolar constraint on surface drag force for various porosity constraint values. It is detected from the figure that surface drag force is less in case of nanoparticles without aggregation than with aggregation. This is because the existence of a porous parameter causes drag on the fluid flow, and an increase in the micropolar constraint increases the boundary layer thickness. As a result, surface drag force declines. Figure 8 exhibit the influence of micro-rotation parameter on wall couple stress factor for various values of micropolar constraint. It is seen from the figure that the rise in the values of micro-rotation parameter and micropolar constraint will enhances the wall couple stress in the system. This is due to enhancement in the vortex viscosity factor.

Figure 9 displays the consequence of solid volume fraction ϕ over a temperature distribution rate for numerous values of heat source/sink constraint Hs. Escalating in the values of Hs and ϕ will upraises rate of thermal circulation. Augmentation in the precise values of ϕ will improves the BLT and increased Hs will improves the heat propagation. As a result, rate thermal distribution improves.

Figure 10 depicts the nature of ϕ over a mass transfer rate for numerous values of thermophoretic constraint τ. An increase in ϕ and τ will decrease the rate of mass transmission. As the values of thermophoretic constraint increases, temperature difference gradient also increases, as a result, the particle motion in the system increases. Oppositely, enhancement in the solid volume fraction will enhances the BLT. Due to presence of thermophoretic constraint, the rate of mass transfer diminishes. Figure 11 illustrates the consequence bioconvection Lewis number Lb on density number of motile microorganisms for various values of bioconvection Peclet number Pe. It is observed that, increased values of Pe will enhance the density number of motile microorganisms.

## 5. Concluding Remarks

In the present investigation micropolar nanoliquid flow past a continuous stretching sheet subject to porous medium, HS/S, TPD, and bioconvection is examined. Here, the analysis is made in the presence and absence of nanoparticle aggregation. The system of equations that represents governing equations are transformed into ODEs with utilizing suitable similarity variables and those equations are solved numerically with the aid of computing software. The influence of various dimensionless parameters is studied with respective profiles. The major outcomes of the present investigation are as follows:

- Enhancement in the porous parameter will diminishes the velocity profile due to presence of porous medium which drags the fluid motion.
- For escalating values of $\alpha_{1}$ velocity is more in the presence of aggregation than absence of aggregation in $f^{\prime }$ but, reverse trend is seen in g.
- Improvement in the values of Hs will improve the profiles of temperature and heat transfer rate. Heat transfer gradually increases from heat sink to source.
- Nanoparticles with aggregation have a lower concentration than nanoparticles without aggregation in the presence of thermophoretic parameter.
- Density number of motile microorganisms will be decreased by improved values of Pe.
- Nanoparticle with aggregation is lesser impact in velocity profile but shows more impact in temperature, concentration and bioconvection profiles.

## Figures and Tables

**Table 1:** Thermophysical properties of nanomaterials.

S. No	Properties	Expressions for Nanofluid
01	Viscosity	$\frac{\mu_{nf}}{\mu_{f}}=\left(1-\frac{\phi_{a1}}{\phi_{m1}}\right){\;}^{-2.5\phi_{m1}}$
02	Density	$\frac{\rho_{nf}}{\rho_{f}}=\left(1-\phi_{a1}\right)+\phi_{a1}\frac{\rho_{a1}}{\rho_{f}}$
03	Heat capacity	$\frac{{\left(\rho c_{p}\right)}_{nf}}{{\left(\rho c_{p}\right)}_{f}}=\phi_{a1}\left(\frac{{\left(\rho c_{p}\right)}_{a1}}{{\left(\rho c_{p}\right)}_{f}}\right)+\left(1-\phi_{a1}\right)$
04	Thermal conductivity	$\frac{k_{nf}}{k_{f}}=\frac{2k_{f}+k_{a1}+2\phi_{a1}\left(k_{a1}-k_{f}\right)}{2k_{f}+k_{a1}-\phi_{a1}\left(k_{a1}-k_{f}\right)}$

**Table 2:** Thermo-physical properties of nanomaterial and base fluid.

Properties	Titanium Dioxide	Water
$\rho \left(kg/m^{3}\right)$	4250	997.1
$C_{p}\left(J\;kgK\right)$	686.2	4179
$k\left(W/\;mK\right)$	8.9538	0.613
Pr	-	6.2

**Table 3:** Assessment of Re Cf with published results for sundry values of $\alpha_{1}$ and $n_{1}$ in the neglecting of $A_{1},A_{2}$ and β.

$n_{1}$	$\alpha_{1}$	Present Study
0.0	0.0	−1.0000
-	1.0	−1.3678
-	2.0	−1.6211
-	4.0	−2.0040
0.5	0.0	−1.0000
-	1.0	−1.2246
-	2.0	−1.4140
-	4.0	−1.7319

## Data Availability

Not Applicable.

## Nomenclature

a	Stretching Constant	$n_{1}$	Microrotation Parameter
C	Concentration	$N_{w}$	The density of motile microorganisms at the wall
$C_{w}\&C_{\infty }$	Wall and ambient concentration	$N_{\infty }$	The ambient density of motile microorganisms
Cp	Specific heat	$Nn_{x}$	Density number of motile microorganisms
$C_{f}$	Skin friction	$Nu_{x}$	Nusselt number
$C_{s}$	Wall couple stress factor	Pe	Bioconvection Peclet number
D	Diffusivity	Pr	Prandtl number
$D_{1}$	Fractal index	$r_{a1}$	Radii of aggregates
$f\left(\eta \right)$	Dimensionless velocity profile	$r_{p1}$	Radii of nanoparticle
$g\left(\eta \right)$	Dimensionless microrotation velocity profile	Re	local Reynolds number
Hs	Heat source/sink constraint	Sc	Schmidt number
j	Microinertia density	$Sh_{x}$	Sherwood number
k	Thermal conductivity	T	Temperature
$k_{a1}$	Thermal aggregation conductivity	$T_{r}$	Reference temperature
$K_{1}$	Coefficient of vortex viscosity	$T_{w}\&T_{\infty }$	Wall and ambient temperature
$K_{2}$	Thermophoretic constant	$u_{w}$	Uniform velocity
$K^{*}$	Permeability of porous media	$V_{T}$	Thermophoretic velocity
Lb	Bioconvection Lewis number	u&v	Velocity components
N	The density of motile microorganism	x&y	Coordinate axis
Greek symbols			
$\Omega_{1}$	Spin gradient viscosity	$\alpha_{1}$	Micropolar parameter
β	Porous parameter	τ	Thermophoretic parameter
ν	Kinematic viscosity	σ	Concentration difference parameter
μ	Dynamic viscosity	ρ	Density
$\phi_{m1}$	Extreme volume fraction	$\phi_{a1}$	Effective volume fraction of aggregates
η	Similarity variable	Ψ	Stream function
θ(η)	Dimensionless temperature profile	$\varphi \left(\eta \right)$	Dimensionless bioconvection profile
χ(η)	Dimensionless concentration profile	ϕ	Solid volume fraction
Subscripts			
a1	aggregates	p1	Nanoparticle
f	Fluid	nf	Nanofluid
